# Maize (*Zea mays* L.): A New Host for *Ligustrum witches’* Broom Phytoplasma

**DOI:** 10.3390/pathogens10060723

**Published:** 2021-06-09

**Authors:** Behçet Kemal Çağlar, Serkan Pehlivan, Ekrem Atakan, Toufic Elbeaino

**Affiliations:** 1 Department of Plant Protection, Faculty of Agriculture, Çukurova University, Adana 01330, Turkey; kecaglar@cu.edu.tr (B.K.Ç.); spehlivan@cu.edu.tr (S.P.); eatakan@mail.cu.edu.tr (E.A.); 2 Istituto Agronomico Mediterraneo di Bari (CIHEAM-IAMB), Via Ceglie 9, Valenzano, 70010 Bari, Italy

**Keywords:** maize, phyllody, phytoplasma, 16S rDNA, PCR, RFLP, phylogenetic analysis

## Abstract

In the 2019–2020 growing season, two corn fields located in İmamoğlu town (Adana Province, Turkey) were surveyed following the appearance of phytoplasma-like symptoms on maize plants. A total of 40 samples were collected and tested in first-round and nested PCR using universal primer pairs P1/P7 and R16F2n/R16R2, respectively. All maize-diseased plants reacted positively, whilst no PCR amplifications were obtained from asymptomatic plants. Blast sequence analysis of R16F2n/R16R2-primed amplicons from different maize isolates showed 99.2% to 100% of identity with the 16S rRNA gene of *Ligustrum witches’* broom phytoplasma (LiWBP). To gain additional molecular information on the 16S ribosomal RNA and 23S rRNA intergenic spacer region of LiWBP, not identified previously, the P1/P7-primed amplicons were also sequenced and analyzed. The results show that maize isolates from Turkey share 99.6% to 100% of identity among them, whereas the highest identity found (91%) was with members of groups 16SrII and 16SrXXV (peanut and tea witches’ broom groups, respectively). This distant relationship between LiWBP and members of 16SrII and XXV was also confirmed by RFLP and phylogenetic analyses. This is the first finding of LiWBP on maize in nature, where it was found responsible for phyllody disease of corn plants in Turkey. The additional molecular information acquired in this study on the 16S–23S rRNA intergenic spacer region of LiWBP further corroborates its distant relationship to any other phytoplasma groups.

## 1. Introduction

Maize (*Zea mays* L.) production occupies third place after wheat and barley in Turkey [1]. According to the Turkish Statistical Institute, the estimated cereal output for 2020 is 36.6 million tons, about 7% percent more than the average of the previous five years, including 20.5 million tons of wheat, 8.3 million tons of barley and 6 million tons of corn [1].

Generally, maize is grown everywhere in the country, but the Çukurova region, known in the 1960s and 1970s as the cotton region, now boasts of being the first-ranked corn-producing region in Turkey [2]. In particular, the province of Adana with a production of 819,978 tons of corn constitutes almost 12% of the national production [3], and this important corn production in Adana is due to the favorable soil and climatic conditions of the Çukurova region.

In Turkey, phytoplasmas are considered among the most important pathogens that are limiting the agricultural production at the national level [4]. The recent discovery of many phytoplasma groups (16SrI, II, VI, IX, X and XII) on many crops such as tomato, cucumber, pepper, maize, peach, pear, pomegranates, eggplant, sesame and ornamentals, in different regions of the country [5,6,7,8,9,10,11,12,13], along with their insect vectors, has increased concern about the potential threat of these pathogens in the very short term to come. To the best of our knowledge, only Bermudagrass white leaf phytoplasma has been reported from maize in the country [6,8]. Although many investigations were conducted on hundreds of maize plants from different regions of Turkey, all were unsuccessful in detecting any presence of phytoplasma on this crop (Çağlar B.K., personal communication). Following this apprehension, several visits to maize fields in İmamoğlu town of Adana Province were scattered during the 2019–2020 growing season, following several reports of severe symptoms that appeared on maize plants similar to those induced by phytoplasmas. Therefore, a molecular investigation was conducted to identify the possible presence and involvement of these pathogens in the newly emerged disease, the results of which are reported and discussed hereafter.

## 2. Results

### 2.1. Detection of Phytoplasma in Field-Collected Samples

**Figure 1:**
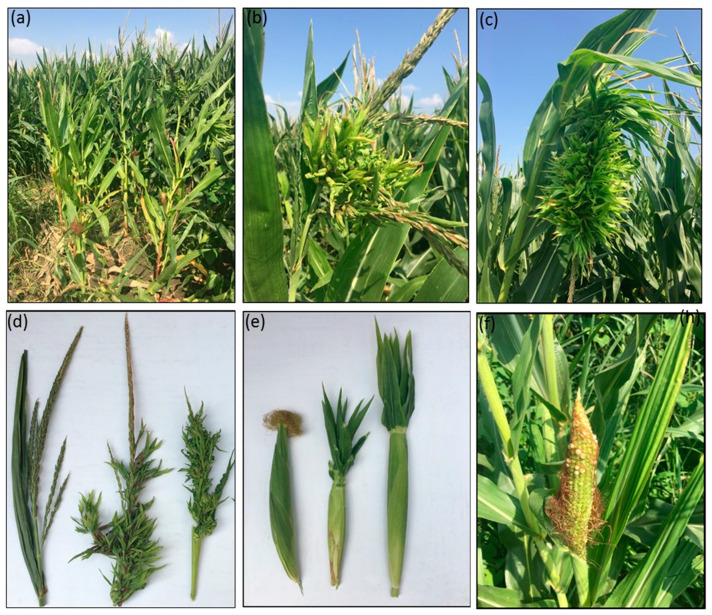
Corn plants showing different types of symptoms observed in the two corn fields visited in the province of Adana. (**a**) Bushy growth and dwarfing of affected plants. (**b**) Formation of multiple inflorescences. (**c**) Witches’ broom-like aspect. (**d**) Healthy male inflorescence (left), phyllody and deformation on tassel of male inflorescence (right). (**e**) Healthy corncob (left), formation of thin, silkless and flag leaf on corncob. (**f**) Seed abortion resulting in empty corncob.

PCR assays conducted on field samples, using R16F2n/R16R2 primers, yielded amplicons from all symptomatic maize plants showing bushy growth, dwarfing, phyllody and small empty corncobs (Figure 1), thus indicating the presence of phytoplasmas.

**Figure 2:**
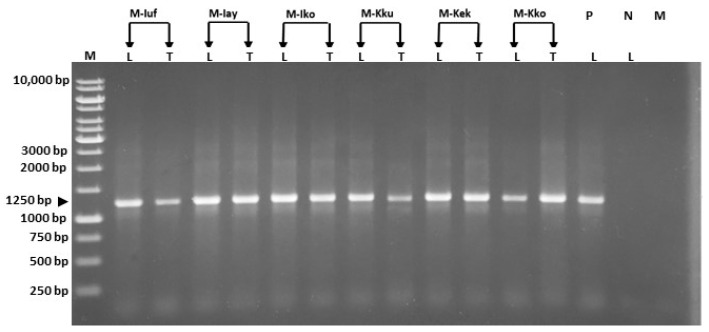
Electropherogram showing R16F2n/R16R2-primed PCR products (1250 bp) obtained from (L) leaf and (T) tassel tissues of diseased maize plants (M-Iuf, M-Iay, M-Iko, M-Kku, M-Kek; M-Kko). P: DNA of *Candidatus* Phytoplasma asteris (16SrI, accession number LR584983) was used as a positive control reaction. N: healthy maize plant; Mix: PCR mix control reaction. Lane M: 1 kb DNA ladder.

In particular, PCR amplicons of the expected 1.25 kbp were generated from both leaf and tassel tissues of diseased maize plants, whereas no amplicons were obtained from asymptomatic plants (Figure 2).

Blast nucleotide sequence analyses of twenty R16F2n/R16R2-primed PCR products, obtained from 10 infected plants/field, showed that the phytoplasma affecting maize plants is the *Ligustrum witches’* broom phytoplasma (LiWBP), previously named as *Ligustrum ovalifolium* phytoplasma [7], which displayed with the Ligustrum isolate Fe1 (accession number HE649495) 99.2% to 100% of identity in the 16S rRNA gene.

To gain additional molecular information on the 16S rRNA and 23S rRNA intergenic spacer region of LiWBP, not identified previously, the P1/P7-primed PCR amplicons of LiWBP-infected maize samples were further sequenced. In total, 12 P1/P7-primed amplicons were observed in agarose gel electrophoresis, out of 30 generated from the use of R16F2n/R16R2 primers in PCR when applied on infected maize plants.

The sequencing of all P1/P7-primed amplicons showed that four sequence types of LiWBP were present in maize-infected plants that shared 99.6% to 100% of identity among them. These isolates were denoted F1/4, F1/51, F2/10 and F2/57 and their sequences were deposited in GenBank with the accession numbers HG994077–HG994080, respectively. However, the Blast analysis showed that members of groups 16SrII and 16SrXXV (peanut and tea witches’ broom groups, respectively) shared the highest identity (ca. 91%) with the LiWBP maize isolates.

### 2.2. Virtual RFLP and Phylogenetic Analysis

**Figure 3:**
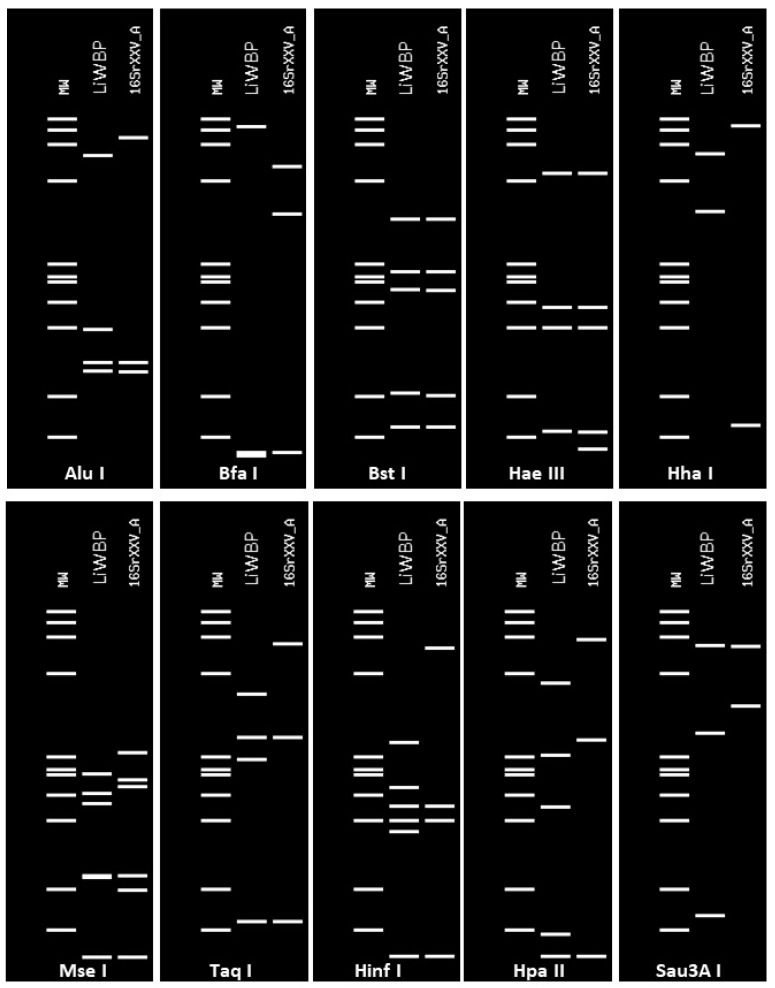
RFLP profiles generated after in silico digestion of 16S rDNA fragment of LiWBP isolate F2/57 from maize (accession number HG994080) compared with the most similar reference pattern of the 16Sr group XXV, subgroup A (GenBank accession: AF521672), with informative enzymes using the online iPhyClassifier.

The virtual restriction fragment length polymorphism (RFLP) patterns derived from the 16S rDNA R16F2n/R16R2 fragments of LiWBP were different from the reference patterns of all previously established 16Sr groups/subgroups (data not shown), but not from LiWBP Fe1 and Fe2 isolates (accession numbers HE649494 and HE649495) previously identified in Ligustrum plants [14]. Although, at the sequence level, the most similar phytoplasma to LiWBP was the weeping tea tree witches’ broom phytoplasma (16Sr group XXV, subgroup A, GenBank accession number AF521672), the virtual RFLP pattern of LiWBP was different from that of the reference strain (Figure 3).

**Figure 4:**
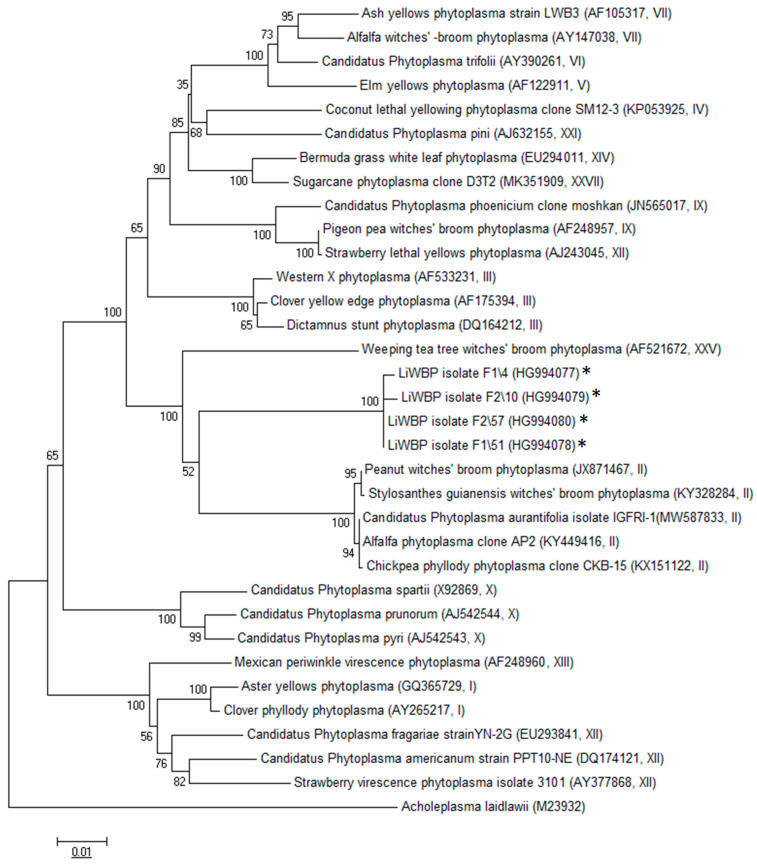
Phylogenetic relationships of LiWBP isolates with the “*Candidatus* Phytoplasma” taxa available. The evolutionary history was inferred using the neighbor joining method [16]. The percentage of replicate trees in which the associated taxa clustered together in the bootstrap test (1000 replicates) is shown next to the branches [17]. The evolutionary distances were computed using the maximum composite likelihood method in MEGA6, using *Acholeplasma laidlawii* as an outgroup, and are in the units of the number of base substitutions per site. Sequences obtained in this study are indicated with an asterisk.

In the RFLP analysis, the similarity coefficient was 0.65, which is much lower than the conventional threshold (0.85) stabilized for the delineation of a new and distinct phytoplasma 16Sr subgroup lineage [15]. This distant molecular relationship of LiWBP with members of groups II and XXV was also corroborated by the phylogenetic tree, which allocated the LiWBP maize isolates together in one cluster distant from members of 16Sr groups II and XXV (Figure 4).

## 3. Discussion

The search for additional phytoplasma-derived sequences different from those found for LiWBP that might be responsible for the disease on maize plants, by sequencing 42 PCR amplicons (30 R16F2n/R16R2- and 12 P1/P7-primed amplicons from infected plants) was unsuccessful, as all sequences found were from LiWBP. These data suggest that LiWBP is a unique pathogen responsible for the symptoms observed on maize-affected plants in the two visited fields. This result was also corroborated by the RFLP analyses (using AluI and HhaI endonucleases) performed on eight R16F2n/R16R2-primed amplicons from infected plants, which generated DNA profiles similar to those obtained from the virtual RFLP for each enzyme (data not shown). The lack of information on phytoplasma infections on maize crops in Turkey makes this study the first report on such infection, together with maize as a new host plant for LiWBP in nature, besides the ornamental California privet plant (*Ligustrum ovalifolium*) and Black locust tree (*Robinia pseudoacacia*) [7,14]. The severe symptoms observed on maize plants and other plant hosts [7] leave no doubt about the seriousness of LiWBP infection, which is gaining more spaces and conquering different plant hosts in Turkey. Accordingly, a timely epidemiological intervention is recommended to cope with the possible expansion of this disease in maize cultivation, which is crucial for the economy of Adana Province.

The search for insect vectors of phytoplasmas, i.e., leafhoppers, planthoppers and psyllid, that might have inoculated LiWBP onto maize plants from other hosts, together with their incidence in maize crops, is ongoing in order to draw an epidemiological scenario of this new pathogen in Adana Province. The sequence information acquired in this study on the 16S–23S rRNA intergenic spacer region of LiWBP suggests its classification as a new taxon among phytoplasma groups, since the 91% of identity found between LiWBP and the closest members of phytoplasma groups (16SrII and 16SrXXV) is largely distant from the demarcation criteria recommended by the Phytoplasma/Spiroplasma Working Team Phytoplasma Taxonomy Group [18], according to which a “*Candidatus* Phytoplasma” species should refer to a single, unique 16S rRNA gene sequence (>1200 bp), and a strain can be recognized as a novel “*Ca.* Phytoplasma” species if its 16S rRNA gene sequence has <97.5% similarity to that of any previously described “*Ca.* Phytoplasma” species.

## 4. Materials and Methods

### 4.1. Sample Collection

During May–July 2019–2020, several visits were conducted in two maize fields (F1 and F2) at İmamoğlu town to investigate the etiology of maize-diseased plants showing symptoms of phyllody and deformation of tassel of male inflorescence, formation of multiple empty corncobs and proliferation in female inflorescence resulting in silkless and empty corncobs, bushy growth and dwarfing (Figure 1). Twenty samples were collected from each field, 15 of which were from symptomatic plants, while the other 5 were apparently healthy. Upon arrival of the samples in the laboratory, the total nucleic acids were extracted from the plant tissues and subjected to molecular analysis (PCR) for the specific detection of phytoplasmas suspected of being responsible for the disease observed in the fields.

### 4.2. DNA Extraction

DNA was extracted from midrib tissues of leaves and tassels of symptomless and diseased plants showing different types of symptoms (Figure 1), according to Ahrens and Seemüller with some modification [19]. Tissue samples of midrib (1 g) were homogenized in 4 mL of CTAB buffer (2% *w*/*v* cetyltrimethylammonium bromide, 1.4 M NaCl, 0.2% 2-β-mercaptoethanol, 20 mM EDTA, 100 mM Tris-HCl, 2% polyvinylpyrrolidone, pH 8.0). Aliquots of 600 μL of the extract were incubated at 65 °C for 30 min. An equal volume of chloroform-isoamyl alcohol (24:1) was added to the sample and vigorously mixed and centrifuged at 12,000 rpm for 10 min. Further extraction was performed with chloroform-isoamyl alcohol (24:1) addition followed by centrifugation at 12,000 rpm for 10 min. The nucleic acid was precipitated overnight at −20 °C in isopropanol, and the pellet was washed with 70% ethanol, dried at room temperature, dissolved in 50 μL of sterile water and used as DNA template for PCR amplification.

### 4.3. Polymerase Chain Reaction (PCR)

Polymerase chain reaction and nested PCR assays were performed using the universal phytoplasma primer pairs P1/P7 [20,21] and R16F2n/R16R2 [22,23], respectively. The P1/P7 primer pair amplifies a PCR product of 1.8 Kbp in size that extends from the 16S rDNA to the 5′ region of the 23S rDNA, whereas the primer pair R16F2n/R16R2 amplifies an internal DNA fragment of 1.25 Kbp. PCR amplification was performed in 50 μL reaction mixtures, each containing 1 μL (50 ng) of extracted DNA, 1 μL dNTPs (10 mM), 1 μL forward and 1 μL reverse primer (10 pmoL), 5 μL of 10X Takara La Taq buffer, 0.25 μL Takara La Taq DNA polymerase (5 U/μL) (Takara Bio Inc., Shiga, Japan) and 40.75 μL sterile distilled water. PCR was conducted in a MiniAmp Plus Thermal Cycler apparatus, Thermo Fisher Scientific (Waltham, MA, USA), using the following parameters: 35 cycles of 1 min at 95 °C, 2 min at 50 °C and 3 min at 72 °C. PCR conditions for the nested PCR were the same, except for the annealing temperature that was at 55 °C. An extension cycle consisting of 10 min at 72 °C was used for both PCRs. An amount of 10 μL of PCR products primed with R16F2n/R16R2 was electrophoresed in 1% agarose gel in 1X TAE buffer using the 1 kb DNA as marker (Thermo Fisher Scientific, Waltham, MA, USA), stained with ethidium bromide and photographed under a high-performance UV transilluminator-UVP (312 nm).

### 4.4. Sequencing, Virtual RFLP and Phylogenetic Analyses

The P1/P7- and R16F2n/R16R2-primed PCR products obtained from maize plants were cloned using StrataClone^TM^ PCR Cloning vector pSC-A (Stratagene, La Jolla, CA, USA), subcloned into *Escherichia coli* DH5α or SoloPACK cells and sequenced from both forward and reverse plasmid (pUC18) sequencing primers (T3 and T7) (Macrogen Europe, Amsterdam, The Netherlands). Four DNA clones from each PCR-positive sample were sequenced. The nucleotide sequence data were assembled by employing the Contig Assembling program of the sequence analysis software BIOEDIT, version 7.0.0 (http://www.mbio.ncsu.edu/Bioedit/bioedit.html, accessed on 22 February 2021).

Sequences of the R16F2n/R16R2-primed PCR products obtained from diseased maize plants were subjected to virtual RFLP analysis using the iPhyClassifier tool [24]. Each 16S rDNA consensus sequence obtained was in silico digested with 17 restriction endonucleases. The generated virtual RFLP profiles were compared with available representative of 16S rRNA groups/subgroups, and the similarity coefficient was calculated.

A phylogenetic tree was constructed using the sequences of 29 “*Candidatus* Phytoplasma” species available in GenBank. The evolutionary distances were computed using the maximum composite likelihood method [25]. The evolutionary history was inferred using the neighbor joining method [16].

## Data Availability

Sequence data reported in this study can be found at the public site of the European Nucleotide Archive database (ENA).
